# The mechanisms of HER2 targeted ADCs are dependent on Rab GTPases

**DOI:** 10.1177/17588359251332473

**Published:** 2025-04-24

**Authors:** Astrid Medhus, Kay Oliver Schink, Ane Sager Longva, Olav Engebraaten, Kristian Berg, Anette Weyergang

**Affiliations:** Institute for Cancer Research, Norwegian Radium Hospital, Oslo University Hospital, Oslo, Norway; Institute for Cancer Research, Norwegian Radium Hospital, Oslo University Hospital, Oslo, Norway; Institute of Basic Medical Science, University of Oslo, Oslo, Norway; Institute for Cancer Research, Norwegian Radium Hospital, Oslo University Hospital, Oslo, Norway; Institute for Cancer Research, Norwegian Radium Hospital, Oslo University Hospital, Oslo, Norway; Department of Oncology, Oslo University Hospital, Oslo, Norway; Institute of Clinical Medicine, University of Oslo, Oslo, Norway; Institute for Cancer Research, Norwegian Radium Hospital, Oslo University Hospital, Oslo, Norway; Department of Pharmacy, University of Oslo, Oslo, Norway; Institute for Cancer Research, Norwegian Radium Hospital, Oslo University Hospital, Ullernchausseen 70, 0379 Oslo, Norway

**Keywords:** ADC, breast cancer, HER2, mechanism of action, RAB5, T-DM1, T-DXd

## Abstract

**Introduction:**

In the era of personalized cancer therapy, antibody-drug conjugates (ADCs) have become one of the fastest-emerging groups of anticancer drugs. ADCs consist of an antibody coupled to a cytotoxic payload by a chemical linker, designed to be cleaved off intracellularly. Understanding the intracellular trafficking and processing of ADCs is crucial for elucidating their mechanism of action.

**Objective:**

This study aimed to compare trastuzumab deruxtecan (T-DXd) to ado-trastuzumab emtansine (T-DM1) with emphasis on Rab GTPase-regulated intracellular trafficking and its impact on ADC efficacy.

**Methods:**

The efficacy of T-DXd and T-DM1 was assessed in a panel of HER2-positive cell lines. Correlations between ADC efficacy and the expression of HER2 and Rab GTPases were evaluated. Functional studies, including knockdown (KD), overexpression, and microscopy, were performed to evaluate the impact of Rab GTPases on ADC cytotoxicity.

**Results:**

In contrast to T-DM1, T-DXd efficacy was found not to correlate to HER2 expression in a panel of HER2-positive cell lines. However, a correlation to RAB5A expression was found for T-DXd efficacy, although not as strong as for T-DM1. Altering the expression of RAB5 in our model system confirmed RAB5 to have an impact on both T-DXd and T-DM1 cytotoxicity, but more on T-DM1. In addition, RAB4a was found to influence T-DXd sensitivity, but not T-DM1, indicating differences in intracellular processing between T-DXd and T-DM1.

**Conclusion:**

The study demonstrates that ADC design significantly influences intracellular trafficking and processing. The linker design, in particular, plays a major role in determining the intracellular fate of an ADC.

## Graphical abstract

**Graphical abstract:**
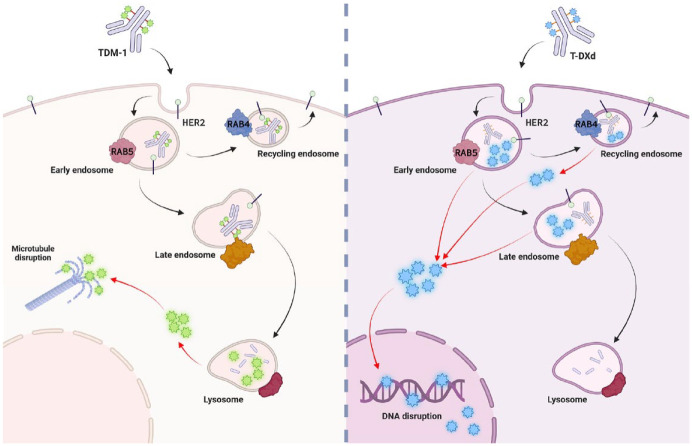

## Introduction

Within the era of personalized cancer therapy, antibody-drug conjugates (ADCs) have become one of the fastest-growing types of anticancer drugs, which has been primarily enabled by the increased knowledge of potential targets, payloads, linkers, and indications.^1^ ADCs consist of a monoclonal antibody coupled to a cytotoxic payload through a chemical linker. The antibody provides a targeted cytotoxic therapy that may spare normal tissue and widen the therapeutic window.^2,3^

The three components of an ADC allow for a wide diversity of compounds combining the target antibody, chemical and pharmaceutical properties of the cytotoxic payload, and the characteristics of the linker that connects the two. These components decide the mechanism of action of each specific ADC, which, in contrast to the naked antibody, exerts its activity inside the cell, after internalization and intracellular processing.^1–4^

Upon internalization, ADCs are trafficked through the endosomal pathways, which are organized by a class of small molecular switches named Rab GTPases.^5^ Over 60 Rab GTPases have been identified in humans. Many of these function as regulators of vesicle trafficking and act as molecular switches cycling between an “on” GTP-bound state and an “off” GDP-bound state.^6^ In their “on” state, they can interact with specific downstream effector molecules and by this recruit specific proteins that determine the identity and consequently the fate of vesicles. As key regulators of intracellular vesicle trafficking, these proteins may be of significance for regulating trafficking and subsequently ADC efficacy.^6^ In line with this, RAB5A, an early endocytic marker, has recently been described as a predictive biomarker for the HER2-targeted ADC ado-trastuzumab emtansine (T-DM1).^7^ However, little is generally known about what impact Rab GTPases have on the therapeutic outcome of other ADCs.

After uptake of an ADC, the cytotoxic payload needs to be released and escape from the endo/lysosomal pathway to exert its intracellular activity. The mechanism of this escape is dependent on the linker, classified as non-cleavable or cleavable.^4,8,9^ Non-cleavable linkers require that the ADC is trafficked to the lysosome where the antibody and linker are degraded, and the cytotoxic payload is released. Cleavable linkers are constructed to release the payload by acidic hydrolysis and/or proteolytic degradation of the linker, resulting in payload release from a large fraction of the endo/lysosomal compartments, independent of lysosomal accumulation.^4,8,9^

The early development of ADCs has been highly focused on HER2-positive breast cancer. HER2 is a transmembrane tyrosine kinase regulating cell growth, survival, and differentiation, and has been linked to poor prognosis and a higher risk of recurrence.^10^ Three out of seven clinically approved ADCs for solid cancer are targeting HER2, representing a novel treatment option for these patients.^11,12^ T-DM1, consisting of the HER2 targeting antibody trastuzumab, a non-cleavable linker (thioether (N-maleimidomethyl cyclohexane-1-carboxylate (MCC)), and the microtubule inhibitor emtansine, was the first clinically approved ADC for solid cancer in 2013.^13–16^ Trastuzumab deruxtecan (T-DXd), also based on trastuzumab, but with a cathepsin-cleavable linker (tetrapeptid) to the topoisomerase I inhibitor deruxtecan, was approved in 2019.^17–19^ The drug-to-antibody ratio (DAR) varies between the different ADCs. T-DM1 has an average DAR of 3.5 compared to an average of 8 for T-DXd.^2^ T-DXd has demonstrated efficacy in HER2 progressing on T-DM1 and has been shown to be effective and also approved for the treatment of breast cancer patients with low HER2 expression.^18,20^

The aim of this study was to investigate and compare the mechanism of action of T-DM1 and T-DXd with respect to internalization and early intracellular trafficking. Here we show that T-DXd and T-DM1 are dependent on different Rab GTPases as a part of their mechanism of action, but are both trafficked through RAB5-positive vesicles with an impact on efficacy.

## Materials and methods

### Cells and culturing

The experiments were performed in five HER2-positive cell lines originally obtained from American Type Culture Collection (ATCC, Manassas, VA, USA); breast cancer cell line SK-BR-3 (HTB-30, ATCC), AU565 (CRL-2351, ATCC), HCC1954 (CRL-2338, ATCC), and MDA-MB-453 (HTB-131, ATCC), and the ovarian cancer cell line SKOV-3 (HTB-77, ATTC). Cells were cultured as previously described.^7^

### Cytotoxicity experiments

Cells were seeded and incubated with T-DXd (Enhertu^®^, DS-8201; Daiichi Sankyo, Tokyo, Japan) or paclitaxel for 72 h, and irinotecan for 120 h, as described previously for T-DM1 (Kadcyla^®^; Genentech, San Francisco, CA, USA).^7^ Longer incubation time for irinotecan is chosen as this drug takes longer to show cytotoxicity compared to the other drugs used in this study. In total, 120 h incubation would not alter the toxicity of the remaining drugs. The relative survival of the cells was plotted against the drug concentration using the SigmaPlot extended graph analysis program 15.0 (Systat Software, Palo Alto, CA, USA), and IC_50_ values were determined by the software.

### Correlation analysis and calculation of enhancement factors

Protein expression of HER2, RAB5, and RAB4 was previously published in Engebraaten et al.^7^ and can be accessed in the source data provided there. T-DXd sensitivity for each cell line, represented as log transformation of 1/IC_50_ values (concentration in nM where 50% cell viability is inhibited), was plotted against the cellular protein expression in SigmaPlot extended graph analysis 15.0. Enhancement factor of T-DM1 and T-DXd was found by comparing irinotecan and paclitaxel IC_50_ values to T-DXd and T-DM1 IC_50_ values, respectively. MDA-MB-453 did not reach IC_50_ with the applied concentrations and was set to 1000 nM during analysis. It was verified that increasing the IC_50_ value beyond this concentration had only a minor impact on the correlation analysis. Correlation was assessed with the SigmaPlot software using linear regression analysis.

### Cellular localization of T-DXd and T-DM1 in SK-BR-3 fixed cells

Cellular localization of T-DXd (Daiichi Sankyo) and T-DM1 (Genentech) was compared in SK-BR-3 cells. A total of 50,000 cells/well were seeded out on coverslips in 48-well plates. After 24 h, cells were incubated with 10 nM of T-DXd or T-DM1 for 15 min, followed by a 2 h chase at 37°C. Cells were fixed in 4% paraformaldehyde (#15714; Electron Microscopy Sciences, Hatfield, PA, USA) in phosphate buffered saline (PBS) for 15 min at room temperature. T-DXd and T-DM1 were detected by anti-human Alexa Fluor (AF) 488 (donkey, #709-545-146; Jackson ImmunoResearch, Ely, UK) staining for 30 min in PBS (#10010056; ThermoFisher (Gibco), Waltham, MA, USA) with 0.05% saponin (#S7900, Sigma-Aldrich, St. Louis, MO, USA) before they were mounted in ProLong™ Glass Antifade Mountant (#P36980). Cells were imaged on a Nikon ECLIPSE Ti2-E inverted microscope (Nikon Corporation, Tokyo, Japan) equipped with a CrestOptics X-Light V3 Spinning Disk confocal (50 µm pinhole) and a Photometrics Kinetix camera. The acquisition was done using a 100× objective (*NA 1.45*, CFI Plan Apo λ D) and a Lumencor Celesta laser unit (477 nm (GFP) emission filter 511/20 nm). Images were processed in Fiji.

### Cell survival analysis after co-incubation with trastuzumab and T-DXd or T-DM1

SK-BR-3 cells were seeded at 8 × 10^3^ in 96-well plates (Nunc, Roskilde, Danmark). T-DM1 and T-DXd solutions were placed in a medium for 24 h at 37°C. After 24 h, cells were pre-incubated with 30 nM trastuzumab (Herceptin^®^; Roche, Basel, Switzerland) for 30 min, before they were co-incubated with 100 nM trastuzumab (Roche), and the T-DM1 or T-DXd solutions for 72 h. The medium was then removed, and the cells were incubated with 0.15 mg/ml MTT (Sigma-Aldrich) for 1.5–2.5 h before the media was removed, and the formazan crystals were dissolved in DMSO. Absorbance was measured at 570 nm using a plate reader (PowerWave XS2 Microplate Spectrophotometer; Biotek, Winooski, VT, USA) and Gen5 software version 2.09 (Biotek). The relative survival of the cells to trastuzumab treatment alone was plotted against the drug concentration using the SigmaPlot extended graph analysis program 15.0 (Systat Software, Palo Alto, CA, USA).

### SiRNA knockdown procedure

siRNA knockdown (KD) of protein expression was performed in SK-BR-3 cells. Cells were seeded out at 8 × 10^3^ cells/well in 96-well plates and at 15 × 10^4^ cells/well in 12-well plates for harvesting western blot samples. Cells were left for at least 4 h for attachment before they were transfected with Lipofectamine RNAiMAX transfection reagent (ThermoFisher Scientific, Waltham, MA, USA) and siRNA (end concentration of 0.25 nM for each siRNA) in Opti-MEM medium (ThermoFisher Scientific) according to manufacturer’s protocol. For Rab5 KD siRNA targeting all three isoforms of RAB5 (A, B, and C) were used in combination. siRNA against RAB4A (#S100301581), RAB5A (#S100301588), RAB5B (#S102662800), RAB5C (#S102663073), and siRNA control (#S103650353) were purchased from Qiagen (FlexiTube siRNA, Hilden, Germany). Twenty-four hours after transfection, the medium was changed to McCoy’s 5A (with serum, Sigma-Aldrich), and the transfected cells were subjected to ADC cytotoxicity evaluation or evaluation of transfection efficacy using western blotting. For cytotoxicity evaluation, cells were incubated with 1 nM T-DXd or 0.1 nM T-DM1 and left for 72 h before cell viability was measured by MTT.

Western blotting and immunodetection were used to verify KD in the transfection experiments. Samples for western blotting were collected in parallel with the viability evaluation for each experiment and performed as previously described.^7^ Twenty-six-well (#5678105) or 15-well (#4568106) SDS-PAGE (criterion TGX Stain-Free gels, (Bio-Rad, Hercules, CA, USA)) were used and a picture of the gel before transfer and the subsequent blot was taken with the ChemiDoc™ densitometer (Bio-Rad) for total protein quantification. Protein expression was detected by primary antibody RAB5A (#PA5-29022; 1:2000) and RAB4A (#PA3-912; 1:1000; ThermoFisher Scientific), and secondary antibody HRP-linked α-rabbit (1:2500; #7074; Cell Signaling Technology, Danvers, MA, USA). Supersignal West Dura Extended Duration Substrate (ThermoFisher Scientific) and ChemiDoc™ densitometer (Bio-Rad) were used for the detection of protein bands. ImageLab 4.1 (Bio-Rad, software) was used for the quantification and RAB protein expressions were correlated to total protein expression.

### Generation of mCherry-tagged Rab5a in SK-BR-3 cells

To visualize Rab5 localization in targeted cells, SK-BR-3 cells were genetically modified to stably express mCherry-Rab5. A lentiviral vector expressing mCherry-tagged Rab5 under the control of the weak PGK promoter was generated. VSV-G pseudotyped lentiviral particles were generated using a third-generation packaging system (Addgene plasmids (Watertown, MA, USA) #12251, #12253, #12259). To achieve low expression, cells were transduced with low virus titers (multiplicity of infection <1). The virus titer was increased to generate cells overexpressing RAB5a. Stable-expressing populations were generated by antibiotic selection.

### Trastuzumab staining

Trastuzumab (Roche) was labeled with Alexa Fluor 568 (#A10238) and Alexa Fluor 488 (#A10235) using Alexa Fluor™ Protein Labeling Kits from Invitrogen (ThermoFisher Scientific), according to the instructions provided.

### Flow cytometry and fluorescence microscopy of trastuzumab-Alexa568 treated cells

SKBR3 cells were seeded in a 6-well plate at 300,000 cells/well for flow cytometry, and 50,000 cells/well for microscopy samples. Cells were subjected to KD of RAB5 as described above. After 24 h the microscopy samples were moved to a µ-Slide 8 Well high dish (Ibidi, #80806, Gräfelfing, Germany). Forty-eight hours after transfection, cells were treated with 25 nM of trastuzumab-Alexa568 for 15 min, washed 4 times with pre-warmed medium, and left for 2 h chase at 37°C before they were subjected to flow cytometry or fluorescence microscopy. For flow cytometry, cells were detached with trypsin (#T3924; Sigma-Aldrich), centrifuged, and resuspended in PBS with 2% FBS before being measured on LSR II flow cytometer (BD Biosciences, Franklin Lakes, NJ, USA) using excitation with a 561-nm laser line. Cells prepared for microscopy were subjected to medium change to FluroBrite DMEM (#A1896701; ThermoFisher Scientific) supplemented with 10% fetal calf serum, 25 mM HEPES (#H0887; Sigma-Aldrich), and 4 mM L-Glutamine (#G7513; Sigma-Aldrich) and imaged using a Nikon ECLIPSE Ti2-E inverted microscope (Nikon Corporation, Tokyo, Japan) equipped with a CrestOptics X-Light V3 Spinning Disk confocal (50 µm pinhole) and a Photometrics Kinetix camera. The acquisition was done with a 60× objective (*NA 1.42*, CFI Plan Apo λ D), and a Lumencor Celesta laser unit (546-nm laser (RFP), emission filter 595/31). The milieu was kept stable by an Okolab top-stage incubator with temperature control, digital CO_2_ control, and active humidification. Images were processed in IMARIS 9.9.1 (Oxford Instruments, Abingdon, UK).

### Live cell imaging of mCherry-Rab5A SK-BR-3 incubated with trastuzuamb-Alex488

Colocalization of RAB5 and trastuzumab was investigated using an SK-BR-3—mCherry-RAB5a cell line and trastuzumab-Alexa488. A total of 30,000 cells per well were seeded out in µ-Slide 8 Well high (Ibidi, #80806) and left overnight to attach. Before imaging, the medium was changed to FluroBrite DMEM (#A1896701; ThermoFisher Scientific) supplemented with 10% fetal calf serum, 25 mM HEPES (#H0887; Sigma-Aldrich), and 4 mM L-Glutamine (#G7513; Sigma-Aldrich). The cells were incubated with 25 nM of trastuzumab-Alexa488 5 min before imaging was initiated using a Nikon ECLIPSE Ti2-E inverted microscope (Nikon Corporation) equipped with a CrestOptics X-Light V3 Spinning Disk confocal (50 µm pinhole), a Photometrics Kinetix camera, and a Lumencor Celesta laser unit 477 nm (GFP) and 546 nm (RFP) (emission filter at 511/20 and 595/31, respectively) with a 60× objective (*NA 1.42*, CFI Plan Apo λ D). The milieu was kept stable by an Okolab top-stage incubator with temperature control, digital CO_2_ control, and active humidification. Images were taken at three random fields of view in each well captured at a 10-min interval for a total of 3 h. In the Fiji software (Fiji Is Just ImageJ^21^), Alexa488 nm fluorescence was measured within mCherry-RAB5 positive vesicles by using the Renyi Entropy threshold on the Alexa568 channel and subsequently measuring the mean fluorescence within each segment. The intensity of Alexa488 nm was plotted as a function of time using the SigmaPlot extended graph analysis 15.0 program.

### Statistical analysis

All statistical analysis was performed in the SigmaPlot extended graph analysis 15.0 program on experiments run at least three times. The data presented is the average of three independent experiments, unless other indicated in the figure legend. Evaluation of significant differences between the two treatments was done by two-sided Student’s *t*-test. A one-way repeated measures analysis of variance followed by a Bonferroni ad hoc test was used when comparing more than two groups in a dataset. *p* Values ⩽0.05 were considered significant.

## Results

### HER2 positive cell lines display different sensitivity to T-DXd and T-DM1

**Figure 1.**
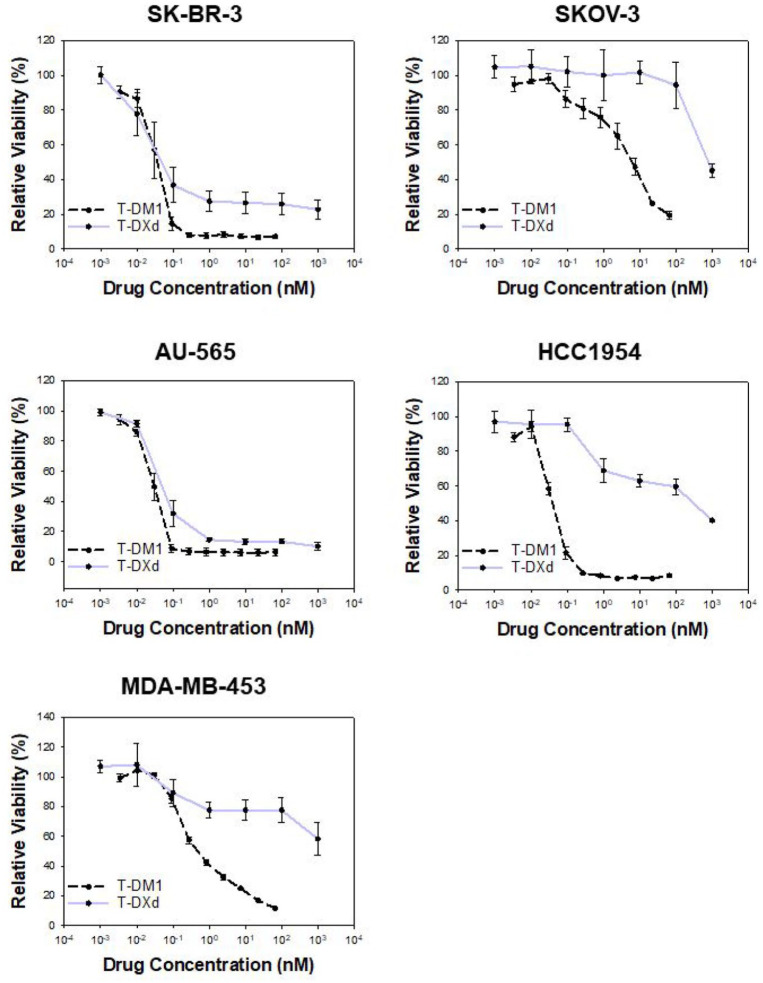
Cellular sensitivity to T-DXd does not follow T-DM1. Relative viability (MTT) of SK-BR-3, SKOV-3, AU-565, HCC1954, and MDA-MB-453 following 72 h treatments with T-DXd and T-DM1. The curves represent the average of the minimum of three independent experiments represented by the data points. Data on T-DM1 is based on source data from Engebraaten et al.^7^ T-DM1, ado-trastuzumab emtansine; T-DXd, trastuzumab deruxtecan.

The *in vitro* response of T-DXd was established in a panel of HER2-positive breast cancer cell lines and compared to the previously reported sensitivity to T-DM1 in the same cell panel.^7^ As T-DM1 and T-DXd differ with respect to cytotoxic payload and DAR, a head-to-head comparison of IC_50_ values in each cell line provides little information on cellular sensitivity. The IC_50_ values for each drug were therefore compared between the cell lines, and the cell lines were identified as highly sensitive (+++), medium sensitive (++), low sensitive (+), or non-sensitive (−) for each drug (Table 1). SK-BR-3 and AU-565 cells were found to be highly sensitive to both treatments, while SKOV-3 responded poorly to both T-DM1 and T-DXd (Figure 1 and Table 1). Notably, the sensitivity to T-DM1 and T-DXd diverged in the HCC1954 and MDA-MB-453 cells. HCC1954 cells were highly sensitive toward T-DM1 (IC_50_ = 0.044 nM), but much more resistant to T-DXd (IC_50_ = 309 nM). MDA-MB-453 was moderately sensitive to T-DM1 (IC_50_ = 0.81 nM) but highly resistant to T-DXd and did not reach IC_50_ with the applied concentrations (Figure 1 and Table 1). Altogether, no consistent pattern between T-DM1 and T-DXd sensitivity was found in the cell line panel.

**Table 1.** Cellular sensitivity of T-DM1, T-DXd, paclitaxel, and irinotecan.

Cell line	T-DXd sensitivity (IC_50_ (nM))	T-DM1 sensitivity (IC_50_ (nM))^a^	Irinotecan sensitivity (IC_50_ (nM))^b^	Paclitaxel sensitivity (IC_50_ (nM))^b^
SK-BR-3	+++ (0.052)	+++ (0.039)	+++ (1129)	++ (3.1)
SKOV-3	+845	− (8.1)	++3664	++3.1
AU-565	+++ (0.053)	+++ (0.031)	++ (2757)	+++ (0.90)
HCC1954	+ (309)	+++ (0.044)	++ (3012)	++ (4.0)
MDA-MB-453	− (1000 EST)	++ (0.81)	+++ ( 1750)	+++ (1.8)

Cellular sensitivity (not sensitive (−), low sensitivity (+), medium sensitivity (++), and high sensitivity (+++)) based on IC_50_ average from three experiments in the indicated cell line. IC_50_ values are collected from the sensitivity plots displayed in Figure 1 and Figure S1. IC_50_ value of MDA-MB-453 was set to 1000 as IC_50_ was not reached. Raw data for T-DM1 sensitivity is gathered from Engerbaaten et al.

a Previously published in Engebraaten et al.^7^

b Corresponding plot published in Supplemental Material.

We tested if the resistance of MDA-MD-435 and HCC1954 cells toward T-DXd could be explained by cellular resistance to the cytotoxic payload of the ADC. SN-38 is the active metabolite of irinotecan and exerts its activity by inhibition of topoisomerase I, like the payload of T-DXd. The sensitivity toward irinotecan was, however, in the same range for all the cell lines, in contrast to the large difference in T-DXd sensitivity in the panel. Likewise, it was studied if T-DM1 sensitivity correlated with paclitaxel sensitivity. Paclitaxel is a microtubule inhibitor and mimics the action of maytansine, the cytotoxic payload of T-DM1. As observed for irinotecan, only small differences in paclitaxel sensitivity were observed within the panel, and no connections were found between T-DM1 and paclitaxel resistance (Table 1 and Figure S1).

### Cellular sensitivity to T-DXd does not correlate to HER2 expression

**Figure 2.**
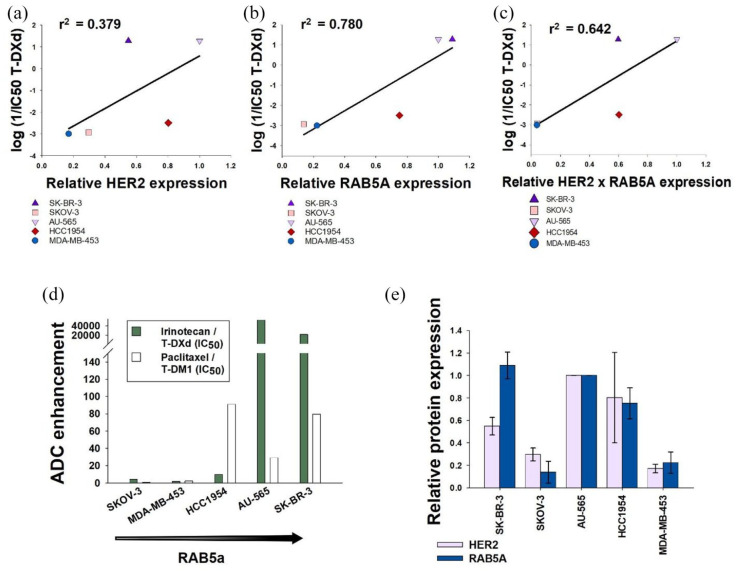
T-DXd sensitivity correlates to RAB5A protein expression, but not HER2. Linear regression analysis curves between T-DXd sensitivity and HER2 (a), RAB5A (b), and HER2 × RAB5A (c) protein expression. The log of the 1/IC_50_ value for each cell line represents the mean of three experiments. Enhancement effect was compared by dividing the IC_50_ of the payload equivalent on the IC_50_ of the relevant ADC (d). Raw data of HER2 and RAB5A protein expression is collected from Engebraaten et al. and quantification of the western blot relative to γ-tubulin is shown in (e).^7^ ADC, antibody-drug conjugates; T-DXd, trastuzumab deruxtecan.

Expression of the target antigen is imperative for the specificity of the ADC, and it was therefore assessed if the cellular sensitivity of T-DXd correlated with expression levels of HER2. There was a large spread in the cellular IC_50_ values compared to T-DM1 values, and a log transformation of the 1/IC_50_ values was done to allow for a better comparison of the data. Nevertheless, expression levels of HER2 could not explain the difference in T-DXd sensitivity among the cell lines, as no correlation (*r*^2^ = 0.379) between T-DXd sensitivity and HER2 expression was found (Figure 2(a) and Table 2). This is in contrast to our previous report on T-DM1 showing a linear correlation between HER2 expression and T-DM1 sensitivity (*r*^2^ = 0.840, Table 2).^7^

**Table 2.** *R*^2^ correlation values between IC_50_ values of T-DXd and T-DM1 and indicated protein expression.

Protein	T-DXd (log (1/IC_50_))	T-DMI (1/1 = IC_50_)^a^
HER2	0.379	0.84
RAB5A	0.78	0.934
HER2 × RAB5A	0.642	0.962

*R*^2^ values from linear regression curves of indicated protein expression and T-DXd and T-DM1 sensitivity. Corresponding T-DXd curves are shown in Figure 2 and T-DM1 data is gathered from Engebraaten et al.

a Previously published data in Engerbaaten et al.^7^

T-DM1, ado-trastuzumab emtansine; T-DXd, trastuzumab deruxtecan.

### T-DXd response correlates to RAB5A expression

T-DXd is dependent on internalization and intracellular processing as a part of its mechanism of action. The correlation between T-DXd sensitivity and HER2 expression could therefore be masked by differences in intracellular trafficking between the cell lines. Rab GTPases are essential regulators of intracellular trafficking, and we have previously shown, both in vitro and in clinical cohorts, that the expression level of RAB5A correlates well with T-DM1 sensitivity.^7^ Thus, it was assessed whether RAB5A expression correlated with T-DXd sensitivity among the cell lines. As shown in Figure 2(b), RAB5A expression was found to have a linear correlation to the T-DXd sensitivity (*r*^2^ = 0.780). Even though the correlation between T-DXd and RAB5A expression was not as strong as previously observed for T-DM1 (*r*^2^ = 0.934, Table 2), it was higher than the one observed for HER2 expression (*r*^2^ = 0.379, Figure 2(a)). It was also evaluated if the combined relative expression of HER2 and RAB5A was a better predictor of sensitivity among the cell lines. However, the data showed a decreased *r*^2^ value (*r*^2^ = 0.642) as compared to RAB5A only (Figure 2(c) and Table 2). This is to some extent in contrast to our previous report, where an increased correlation to T-DM1 response was found when combining HER2 and RAB5A expression levels (*r*^2^ = 0.962, Table 2). Relative protein expression is collected from Engebraaten et al.^7^ and displayed in Figure 2(e).

### The enhancement factor of T-DXd correlates to RAB5A expression

To confirm that the observed correlations were dependent on the cytotoxic component being linked to an antibody, and not the cytotoxic component alone, it was assessed whether the cellular response to irinotecan and paclitaxel correlated to RAB5A expression. However, no correlation was found between the sensitivity toward these payload equivalents and RAB5A expression (Figure S2). As the delivery of payloads via linkage to antibodies intends to enhance the specificity and efficacy of the cancer treatment, the efficacy of T-DXd and T-DM1 was compared to irinotecan and paclitaxel. In contrast to the IC_50_ values for the ADCs, the IC_50_ values of both chemotherapeutics varied only moderately between the cell lines (Table 1). Thus, the enhancement factor by linking the payloads to trastuzumab varied highly between the cell lines (Figure 2(d)). T-DXd enhanced the topoisomerase efficacy by more than 10^4^-fold in the SK-BR-3 and AU-565 cells, while in MDA-MB-435 cells, the enhancement effect was negative (0.087). In T-DXd, a mean of eight molecules of the topoisomerase inhibitor are linked to each antibody, further underlining no enhancement of the topoisomerase inhibitor efficacy after antibody linkage in MDA-MB-435 cells.^2^ The same pattern was seen in the paclitaxel/T-DM1 cohort, where the enhancement effect in the MDA-MB-435 cells was only 2.2 with a mean of 3.5 paclitaxel molecules linked to each antibody.^2^

### T-DXd and T-DM1 show similar cellular localization

**Figure 3.**
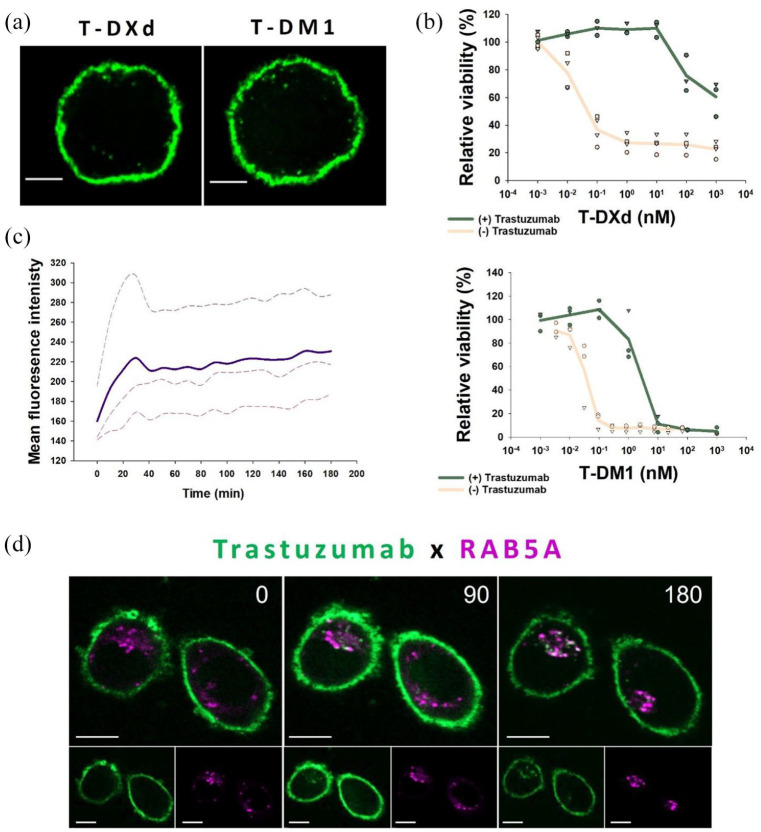
T-DXd and T-DM1 display similar cell binding. Furthermore, AF488-trastuzumab and mCherry-RAB5A colocalize in endocytic vesicles. (a) SK-BR-3 was incubated with 10 nM of T-DXd or T-DM1 and subsequently fixed and detected using an AF488 labeled-anti-human antibody (green). Scale bar = 5 µm. (b) In vitro sensitivity of SK-BR-3 cells to T-DM1 and T-DXd (beige) in combination with 100 nM trastuzumab (green). The ADCs were subjected to 24 h incubation in a cell-free medium prior to incubation, and cell viability was measured after 72 h incubation by MTT. (c,d) Live cell time laps of SK-BR-3—mCherry-Rab5A cells incubated with AF488-trastuzumab. Images were captured in 10-min intervals for 3 h starting 5 min after AF488-trastuzumab incubation. Mean fluorescence intensity of AF488-trastuzumab colocalized with mCherry-RAB5a is shown in (c). The mean is denoted in bold, with each experiment (*n* = 3) shown as stippled lines. Representative sequential images are displayed with the time in minutes shown in the top right corner (d). Scale bar = 10 µm. ADC, antibody-drug conjugates; T-DM1, ado-trastuzumab emtansine; T-DXd, trastuzumab deruxtecan.

The process of conjugating the payload to an antibody may influence the binding specificity and affinity to the target protein. To rule out that the differences in dependence on HER2 for the two trastuzumab-based ADCs were not caused by discrepancies in the localization of the antibody backbone to HER2, SK-BR-3 cells were incubated with T-DXd and T-DM1 for 15 min followed by a 2 h chase. Microscopy showed a strong plasma membrane localization for both ADCs and no apparent difference in cellular localization or trafficking was observed between T-DXd and T-DM1 (Figure 3(a)).

As no differences in cellular localization were observed, it was investigated if the medium (without the presence of cells) caused cleavage of T-DXd. Free payload released by this mechanism could mask a T-DXd sensitivity dependence on HER2 expression. T-DM1 and T-DXd were subjected to 24 h incubation in a medium (without cells) before being added to SK-BR3 in combination with excess trastuzumab (Figure 3(b)). Trastuzumab was shown to inhibit both T-DM1 and T-DXd toxicity, demonstrating a HER2-mediated mechanism of action for both ADCs.

### Trastuzumab localizes to RAB5A-positive vesicles

To confirm that the HER2-targeted ADCs T-DXd and T-DM1 are trafficked through RAB5-positive vesicles, SK-BR-3-mCherry-RAB5a cell lines were incubated with fluorescent trastuzumab. Trastuzumab was found to rapidly localize to the plasma membrane, where it was detected already in the first image captured 5 min after incubation (Figure 3(c) and (d)). Internalization of trastuzumab was observed from around 20 min incubation and throughout the experiment. Plotting the mean fluorescence intensity of trastuzumab-AF488 recorded in mCherry-RAB5 positive vesicles as a function of time, showed a rapid increase in colocalization the first 30 min after trastuzumab incubation, followed by a stable colocalization the following 2 h (Figure 3(c)). Thus, intracellular trafficking of trastuzumab involves a RAB5-positive compartment.

### RAB5A has less impact on cellular response to T-DXd compared to T-DM1

**Figure 4.**
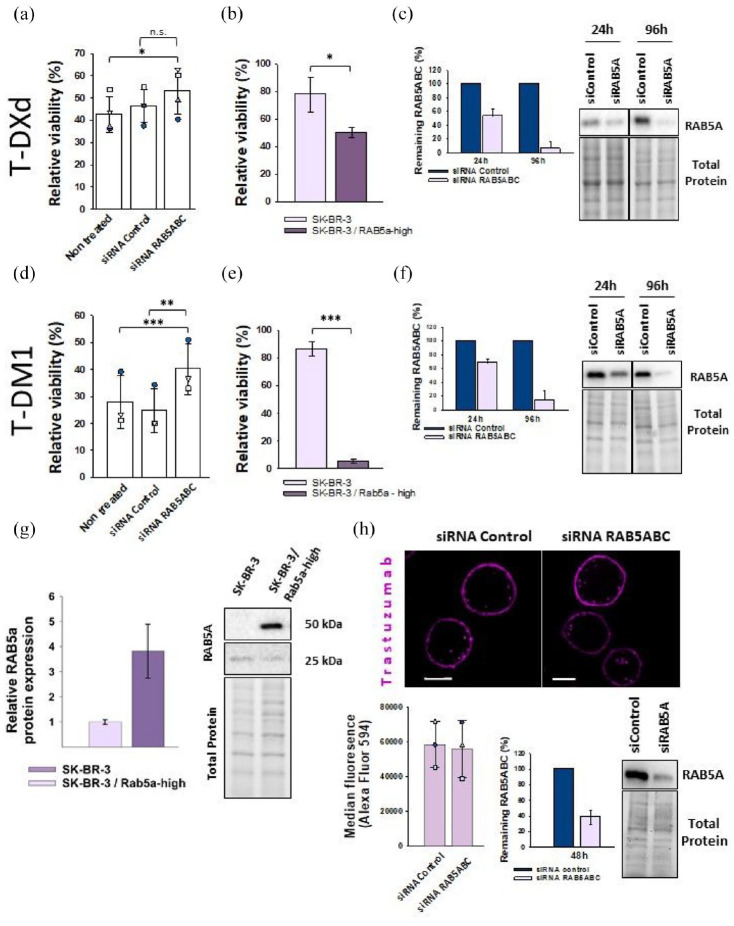
RAB5 impact T-DXd and T-DM1 cytotoxicity but not uptake of trastuzumab. Relative viability (MTT) of cells after KD of RAB5ABC in SK-BR-3 cells and subsequent 72 h treatment of 1 nM T-DXd (a) or 0.1 nM T-DM1 (d). Relative viability is shown with the bars representing the mean of the data and points representing the individual experiments. Statistical significance was determined by a one-way repeated measures analysis of variance followed by a Bonferroni ad hoc test. KD efficiency was verified by western blots and RAB5 was quantified relative to total protein ((c) and (f)). The bars represent the mean of three experiments. Representative western blots are shown and complete blots and full timeline of the KD can be found in Figure S3. (b, e) The relative viability (MTT) of SK-BR-3/Rab5a-high cells after 72 h incubation with 0.01 nM T-DXd or T-DM1. Relative viability is shown with the bars representing the mean of the three indicated data points from each experiment. Statistical significance is determined by a Student’s *t*-test. (g) Western blot and quantification verifying increased RAB5A expression in SK-BR-3/Rab5-high cells. The bars represent the mean of three experiments and representative western blots are shown. (h) RAB5ABC KD in SK-BR-3 cells were verified by western blot and left for 48 h prior to Alexa-568-Transtuzumab incubation (15 min incubation followed by 2 h chase) before they were investigated by flow cytometry and fluorescence microscopy. The KD efficiency at 48 h is presented as the average of three independent experiments and a representative western blot is shown. Median cellular fluorescence of Alexa Fluor 594 is shown as the average of three experiments with each experiment denoted as data points. Representative microscope images are displayed. Error bar: SD. Scale bar = 10 µm. *p ⩽ 0.05, **p ⩽ 0.01. ***p ⩽ 0.001. n.s., *p* > 0.05. ADC, antibody-drug conjugates; KD, knockdown; T-DM1, ado-trastuzumab emtansine; T-DXd, trastuzumab deruxtecan.

The correlation between RAB5A expression and cellular sensitivity to T-DXd and T-DM1 suggests RAB5 is a contributor to the mechanism of action of both ADCs. Thus, SK-BR-3 cells were transfected with siRNA targeting all RAB5 isoforms (RAB5A, RAB5B, RAB5C) and subjected to T-DM1 or T-DXd treatment. The efficiency of the KD increased with time after transfection, and the RAB5ABC expression level decreased from 40%–60% at 24 h to 5%–10% at 96 h (Figure 4(c) and (f)). When treated with T-DXd, siRNA RAB5ABC (siRAB5ABC) SK-BR-3 cells did show an increase in viability compared to the non-treated cells and control cells in 4/4 experiments. The variation in ADC-induced cytotoxicity between the experiments was large and statistical significance was evaluated by a one-way repeated measures analysis of variance where the data are treated as paired within the different experiments. The test revealed a significant difference between the groups (*p* = 0.029). Further evaluation with the Bonferroni ad hoc test revealed a significant difference in viability between RAB5 KD and non-treated cells, but not when compared to control cells (*p* = 0.033 and *p* = 0.179, Figure 4(a)). Statistical significance for differences observed for cells treated with T-DM1 was evaluated as described for T-DXd and revealed a significant difference between the groups (*p* = <0.001), with a significant increase in viability found in siRAB5ABC KD SK-BR-3 cells compared to non-treated and control cells (*p* = <0.001 and *p* = <0.001, Figure 4(d)). In addition, SK-BR-3/RAB5a-high cells, modified to increase the expression level of RAB5a, showed a significantly increased sensitivity toward both T-DXd (two-tailed *t*-test, *p* = 0.409) and T-DM1 (two-tailed *t*-test *p* = 0.000104) at 0.01 nM compared to wild type SK-BR-3 (Figure 4(b), (e), and (g)). Altogether, RAB5 levels appear to have less impact on T-DXd cytotoxicity as compared to T-DM1.

### RAB5 does not regulate uptake of trastuzumab

As both T-DXd and T-DM1 sensitivity were found to correlate to RAB5A expression, and trastuzumab was found in RAB5A-positive vesicles, it was examined whether RAB5 was required for cellular uptake of trastuzumab. Cells were transfected with siRNA control or siRNA against RAB5ABC, resulting in a ~60% reduction in RAB5A expression (Figure 4(h)) and subsequently incubated with Alexa Fluor 568-labeled trastuzumab for 15 min followed by a 2 h chase. Flow cytometry showed no difference in mean fluorescence between control cells and RAB5ABC KD cells (Figure 4(h)). To distinguish between membrane-associated and internalized trastuzumab, we applied fluorescence microscopy to evaluate the cellular localization of trastuzumab in control and RAB5ABC KD cells. However, we detected no difference in intracellular trastuzumab AF-568 levels or cellular localization patterns between the conditions (Figure 4(h)). Thus, we found no evidence that RAB5 regulates the uptake of trastuzumab.

### RAB4 has minor effects on T-DXd efficacy

The difference in the intracellular mechanism for the release of the payload from T-DXd and T-DM1 suggests a different utilization of the endosomal pathway.^15,18^ The tetrapeptide-based linker in T-DXd is cleaved by cathepsins.^18^ These enzymes are present throughout the endo/lysosome system from early endosomes to lysosomes.^22^ High cathepsin activity has previously been reported in RAB4-positive early recycling vesicles and recycling endosomes have been shown to contribute to a substantial part of the processing of cleavable linkers.^23,24^ On the other hand, RAB4 controls the recycling of endocytosed material back to the plasma membrane and could contribute to exocytosis and less ADC-induced toxicity.

**Figure 5.**
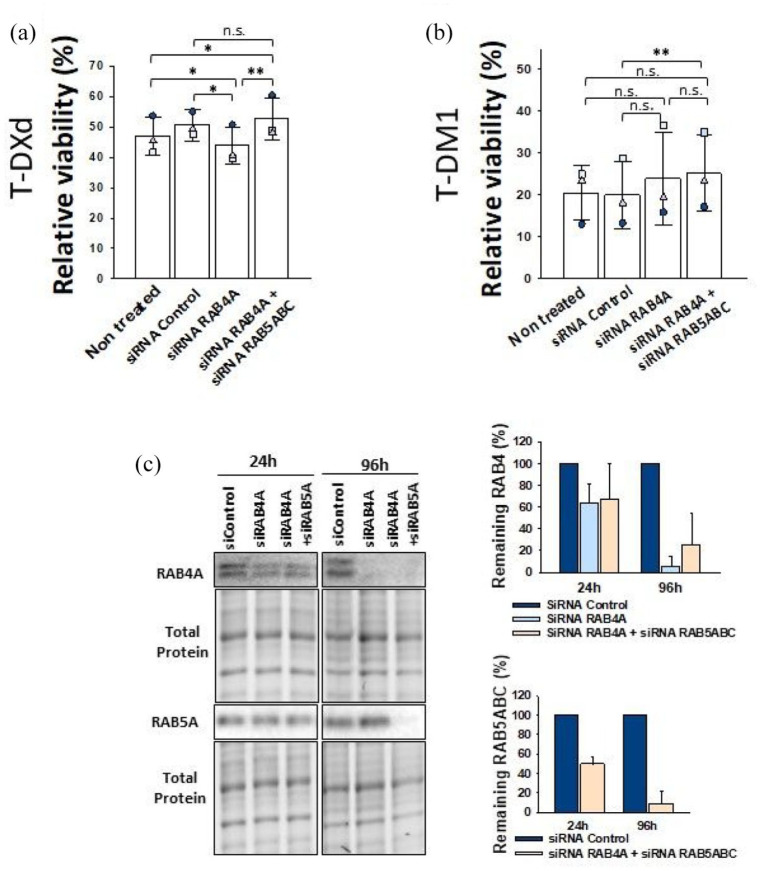
RAB4A impacts T-DXd, but not T-DM1 cytotoxicity. Relative viability (MTT) of SK-BR-3 cells after KD of RAB5ABC and RAB4A and 72 h treatment of 1 nM T-DXd (a) or 0.1 nM T-DM1 (b). Bars represent the mean of three independent experiments indicated as data points. Statistical significance was determined by a one-way repeated measures analysis of variance followed by a Bonferroni ad hoc test. (c) KD efficiency verified by western blots (representative blots shown) quantified relative to total protein. Complete blots can be found in Figure S3. The bars represent the mean of three experiments, and the error bar is the SD. *p ⩽ 0.05. **p ⩽ 0.01. n.s., *p* > 0.05. KD, knock down; T-DM1, ado-trastuzumab emtansine; T-DXd, trastuzumab deruxtecan.

To elucidate the role of RAB4 in T-DXd and T-DM1 trafficking, SK-BR-3 cells were subjected to RAB4 KD by siRNA (Figure 5(a)–(c)). One-way repeated measures analysis of variance test with a Bonferroni post hoc analysis revealed a significant difference between the treatment groups for cells treated with T-DXd (*p* = 0.002) but not T-DM1 (*p* = 0.249, Figure 5(a) and (b)). For T-DXd-treated cells, a weak decrease in survival was found in RAB4 KD cells compared to control cells (*p* = 0.012) but not in non-treated cells (*p* = 0.299, Figure 5(a)). Dual KD of RAB4A and RAB5ABC revealed a minor increased survival compared to siRNA RAB4A KD cells only in SK-BR-3 cells subjected to T-DXd (Bonferroni *t*-test, *p* = 0.003, Figure 5(a)). Comparing the dual KD cells revealed no significant difference for T-DXd efficacy when compared to control cells, however, a small significant change compared to non-treated (Bonferroni *t*-test, *p* = 1 and *p* = 0.032). Altogether, the siRNA data indicate that RAB4 might be involved in the mechanism of action of T-DXd, but not T-DM1 and that RAB4 activity appears to be dependent on RAB5. However, the differences are minor, and it is not clear if RAB4 has biologically relevant impact of RAB4 on T-DXd or T-DM1-induced cytotoxicity in this experimental setup.

## Discussion

The introduction of ADCs has significantly improved the treatment of patients with advanced malignant disease. Cellular targeting and intracellular trafficking are key elements in the mechanism of action of these compounds, and here we report on the intracellular trafficking of two clinically approved HER2-targeted ADCs, T-DXd and T-DM1. Despite the use of the same targeting antibody, we found that while T-DM1 efficacy correlated to cellular HER2 expression, T-DXd efficacy did not (Table 2 and Figure 2). Furthermore, no increased correlation was found between T-DXd sensitivity and the protein expression of both RAB5 and HER2 compared to RAB5 alone (Figure 2(b) and (c) and Table 2). This is in contrast to what was observed for T-DM1 but is in agreement with the HER2 correlation data, where no correlation is found between T-DXd sensitivity and HER2 expression (Figure 2(a) and Table 2). The difference in correlation to HER2 expression could not be explained by resistance toward the cytotoxic payload or differences in HER2 binding or internalization between the ADCs (Table 1 and Figure 3).

In contrast to T-DM1, T-DXd is documented to exert its action partly through bystander activity. T-DXd has previously been shown to have an effect on both HER2-positive and HER2-negative cells in a co-culturing condition, while T-DM1 only had an effect on HER2-positive cells, attributed to its lack of bystander effect.^25^ When the linker of T-DXd is cleaved, it results in a highly lipophilic deruxtecan, able to diffuse not only through the endo/lysosomal membrane but also through the plasma membrane with effect on neighboring cells.^17,18^ Cleaved T-DM1, however, results in the cytotoxic payload being released as positively charged lysine-MCC-DM1, which leaves the endo/lysosomes through specific membrane proteins without the ability to diffuse through the plasma membrane and target other cells.^26,27^ It is, however, difficult to understand how the linker and the lipophilic released payload of T-DXd would mask HER2 dependency in a cell line system, as the processed T-DXd should be proportional to HER2 expression. In agreement with this, pre- and co-incubation with trastuzumab show the mechanism of T-DXd as HER2 specific (Figure 3(b)). On the other hand, cathepsins can be secreted out of the cells, in particular in cancer cells.^22^ A study recently found that extracellular release of cathepsin L in HER2 low breast cancer cells enables cleavage of T-DXds linker and subsequent payload release.^28^ This T-DXd-dependent cytotoxicity is not related to cellular uptake and would therefore not be reliant on the quantity of antigen expression on the cell surface. T-DM1, however, does not harbor a linker that facilitates extracellular cleavage and is consequently dependent on complete internalization and hence HER2 expression.

There is a possibility that insufficient and different linker cleavage between the cell lines could explain the discrepancy between HER2 expression and cellular DXd sensitivity. The tetra peptide linker harbored by T-DXd is cleaved by cathepsins which are often upregulated in tumor cells; however, the cellular expression level and function may differ.^22^ Poor cathepsin function could explain the heightened resistance to T-DXd compared to T-DM1 in HCC1954 and MDA-MB-453. However, other peptide-linker-based ADCs have been previously shown to be effective in these cell lines, indicating a normal cathepsin function.^29,30^ We have not concluded why T-DM1, but not T-DXd, correlates to HER2 expression in the presented panel of cell lines. However, our data is in compliance with clinical data where the response in HER2-positive patients can be correlated to HER2 expression for T-DM1 but not T-DXd.^31,32^

Even though T-DXd efficacy was not found to correlate with HER2 expression, it correlated with RAB5A in accordance with our previous report on T-DM1. RAB5A was found to participate in intracellular transport of trastuzumab, indicating RAB5 to control T-DXd and T-DM1 trafficking, but not uptake (Figures 3 and 4). In agreement with this, a recent study on macropinosome formation reported that RAB5 did not localize to macropinosomes until the vesicles were released from the plasma membrane.^33^ Manipulation of RAB5 expression leads to changed T-DM1 and T-DXd cytotoxicity, indicating a direct connection between RAB5 and T-DXd/T-DM1 response (Figure 4). Furthermore, our results show RAB5 to have a larger impact on T-DM1 as compared to T-DXd cytotoxicity. The mechanism of how RAB5 controls ADC toxicity warrants further investigation. Future experiments could include biochemical assays measuring payload release in the cell line panel.

We also investigated the impact of RAB4, an early recycling marker, on T-DM1 and T-DXd cytotoxicity. Only minor changes were observed for T-DXd and none for T-DM1 (Figure 5(a)). RAB4A KD in combination with RAB5ABC seemed to reverse the small increase in T-DXd sensitivity, suggesting that the presence of T-DXd in RAB4-positive vesicles is partly dependent on trafficking from RAB5-positive vesicles. This is in coherence with findings that internalized cargo moves through RAB5 positive vesicles to RAB4 positive vesicles.^5^ However, the data is weak and more experiments are warranted to conclude if RAB4 has an impact on T-DXd efficacy. Efforts have been taken to generate cell lines with altered RAB4 expression, and also fluorescently tagged RAB4 to aid with further investigation. However, our efforts have not been successful.

## Conclusion

**Figure 6.**
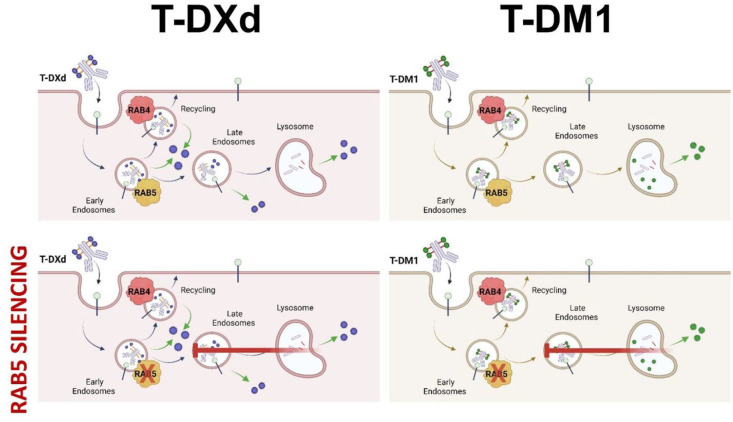
Effect of RAB5 silencing on T-DM1 and T-DXd effect. Graphical suggestion of how RAB5 silencing could affect intracellular trafficking of T-DXd and T-DM1 and hence efficacy. RAB5 silencing can lead to halted or incomplete maturation of endosomes. The effect of T-DXd will be less influenced than T-DM1 due to the nature of the linker. T-DM1 is reliant on trafficking to the lysosome for the payload to escape and have an effect. T-DXd is exposed to cathepsins throughout the endosomal pathway, and the payload will still be able to act upon its target even when endosomal maturation is faulted. Source: Created with BioRender.com. T-DM1, ado-trastuzumab emtansine; T-DXd, trastuzumab deruxtecan.

Overall, our findings reveal differential dependence on intracellular trafficking and processing between T-DXd and T-DM1 with impact on cellular efficacy. If silencing of RAB5 leads to impaired trafficking and early recycling, T-DM1 will not be able to release its payload due to a lack of lysosomal accumulation. T-DXd on the other hand, is not reliant on complete trafficking to the lysosome to have its payload cleaved off and act on the target. This makes T-DM1 more reliant on RAB5 for complete lysosomal trafficking compared to T-DXd (Figure 6). Our results highlight the importance of the interplay between the ADC linker and intracellular trafficking and demonstrate that the linker is an important determinant for payload release and efficacy.

## Supplemental Material

sj-docx-1-tam-10.1177_17588359251332473 – Supplemental material for The mechanisms of HER2 targeted ADCs are dependent on Rab GTPases Supplemental material, sj-docx-1-tam-10.1177_17588359251332473 for The mechanisms of HER2 targeted ADCs are dependent on Rab GTPases by Astrid Medhus, Kay Oliver Schink, Ane Sager Longva, Olav Engebraaten, Kristian Berg and Anette Weyergang in Therapeutic Advances in Medical Oncology

## Declarations

**Ethics approval and consent to participate:** Ethics approval and informed consent were not sought for the present study as no clinical or research animal material was used.

**Consent for publication:** Not applicable.

**Availability of data and materials:** Data will be made available upon reasonable request to the corresponding author.
