# Supplementary material for: The mechanisms of HER2 targeted ADCs are dependent on Rab GTPases
Source: Ther Adv Med Oncol. 2025 Apr 24;17:17588359251332473. doi: 10.1177/17588359251332473 (PMC12035104; doi:10.1177/17588359251332473)
Supplement: sj-docx-1-tam-10.1177_17588359251332473 – Supplemental material for The mechanisms of HER2 targeted ADCs are dependent on Rab GTPases [file sj-docx-1-tam-10.1177_17588359251332473.docx]

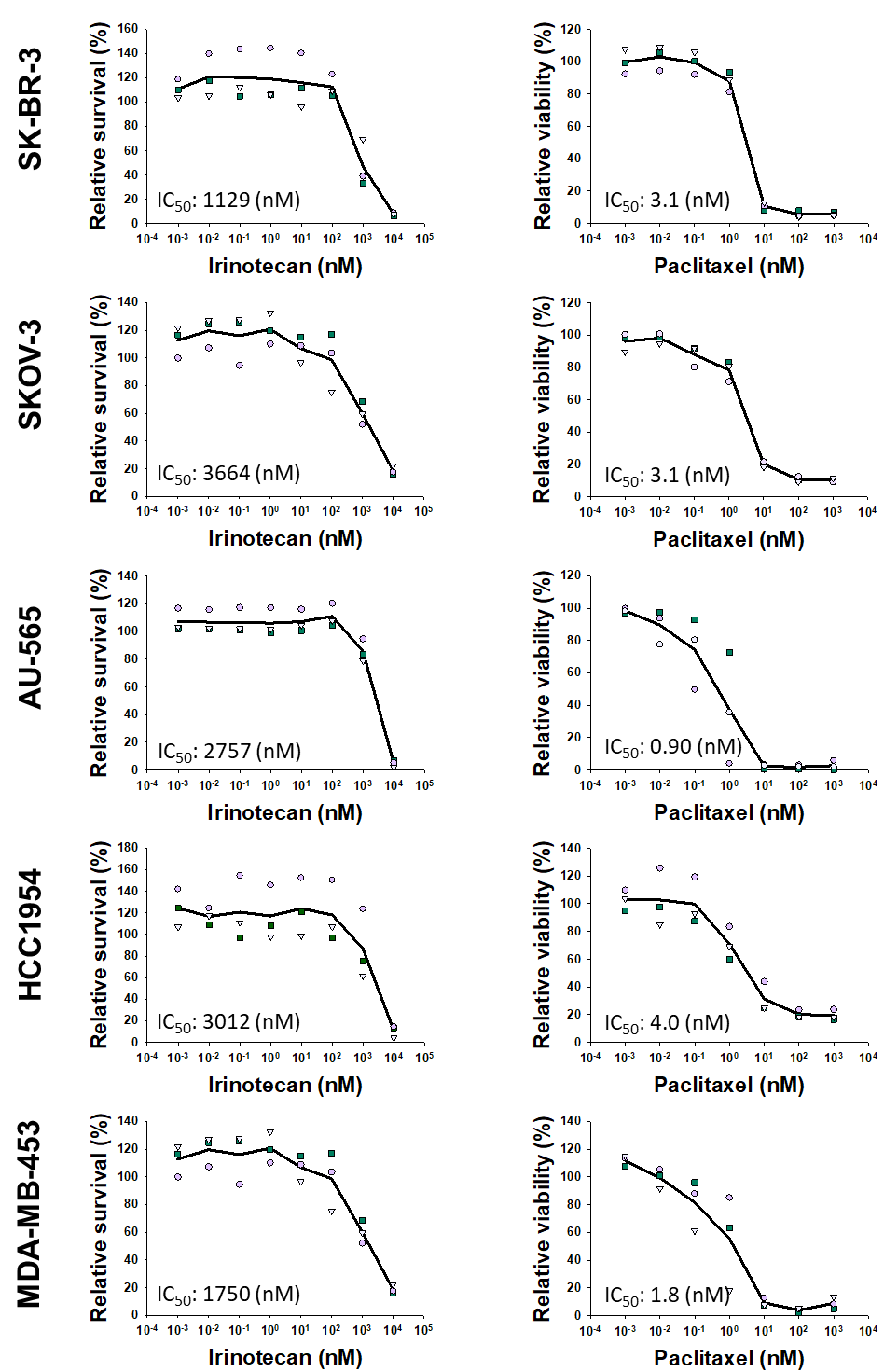


**Figure S1: *Cellular* sensitivity to irinotecan and paclitaxel.** Relative viability (MTT) of SK-BR-3, SKOV-3, AU-565, HCC1954 and MDA-MB-453 following 120 h treatments with irinotecan and 72 h treatment with paclitaxel. The curve indicates the average of minimum 3 independent experiments represented by the data points.


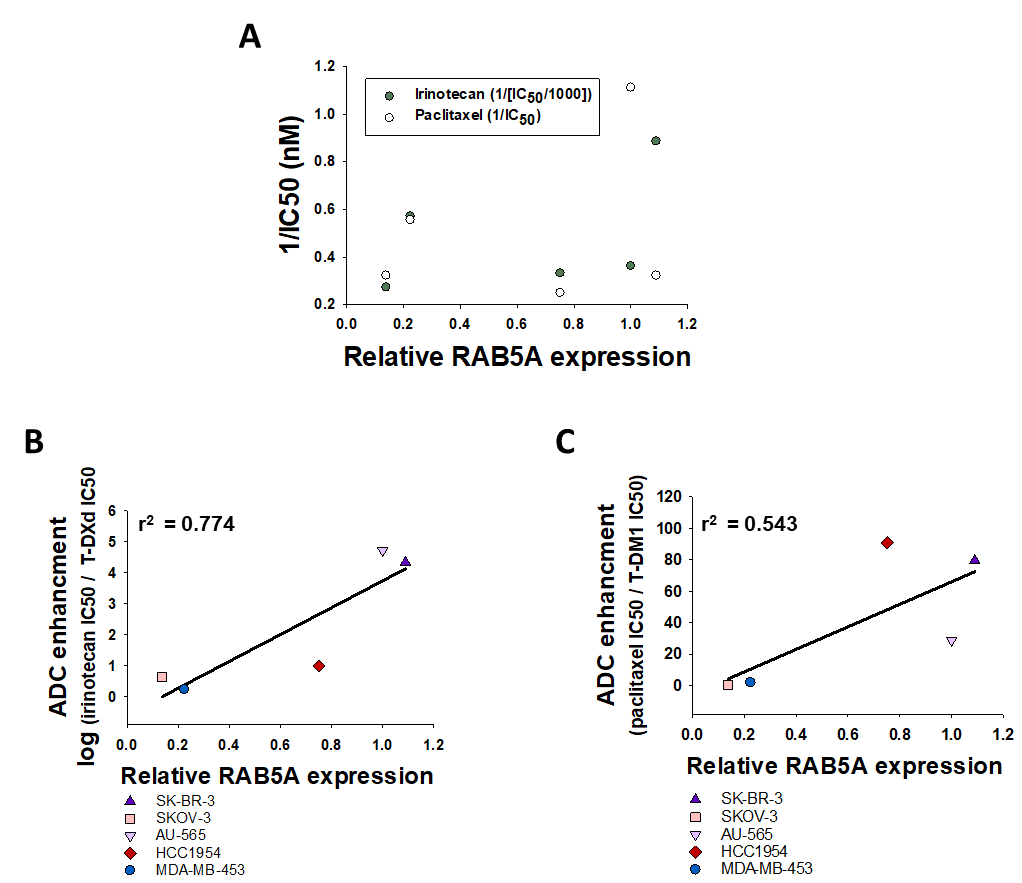


**Figure S5: T-DXd sensitivity correlates the best to RAB4 x RAB5 protein expression**. Linear regression curves between T-DXd and T-DM1 sensitivity and RAB4 expression (A) and RAB4A x RAB5A expression (B). Raw data of RAB5A expression is collected from Engebraaten *et.al*.

**Figure S2: Enhanced sensitivity of T-DXd and T-DM1 as compared to irinotecan and paclitaxel is most prominent in RAB5 high cells.** The 1/IC_50_ values of irinotecan and paclitaxel was plotted against RAB5a expression. 1/IC_50_ value represents the mean of 3 independent experiments. The IC_50_ of Irinotecan was divided by a factor of 1000 for comparison to paclitaxel. No correlation was found between irinotecan sensitivity or paclitaxel sensitivity and RAB5A expression (A). Enhancement effect was compared by dividing the IC_50_ of the payload equivalent on the IC_50_ of the relevant ADC. Linear regression plots between the log transformed enhancement effect of T-DXd (B) or the enhancement effect of T-DM1 (C) and relative RAB5A expression. Raw data of RAB5A expression is collected from Engebraaten *et.al*.


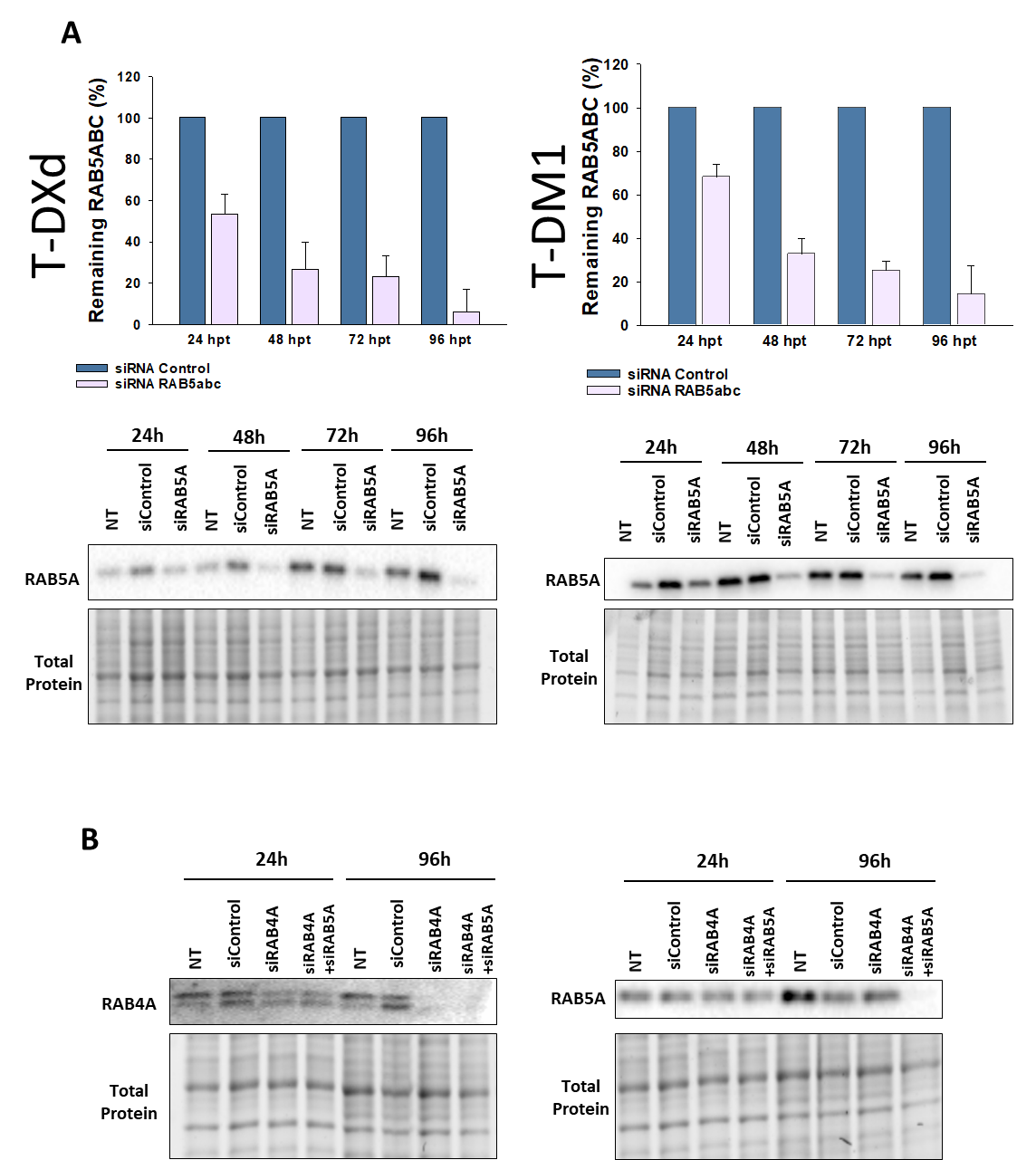


**Figure S3: Knock down efficacy of RAB5ABC in SK-BR-3 over 96 h**. Complete western blots and timeline of the knock down efficiency shown in Figure 4 and in Figure 5. SK-BR-3 cells were subjected to siRNA RAB5ABC transfection and samples were collected at 24, 48, 72 and 96 h and ran on Western blots to review knock down efficacy (A, Figure 4). Samples taken from knock down of RAB4A alone and in combination with RAB5 was run on Western blots to review knock down efficacy (B, Figure 5). Data points represent the quantified western blots normalized to total protein for three individual experiments. The bars represent the mean of three experiments and the error bar the SD of the mean.


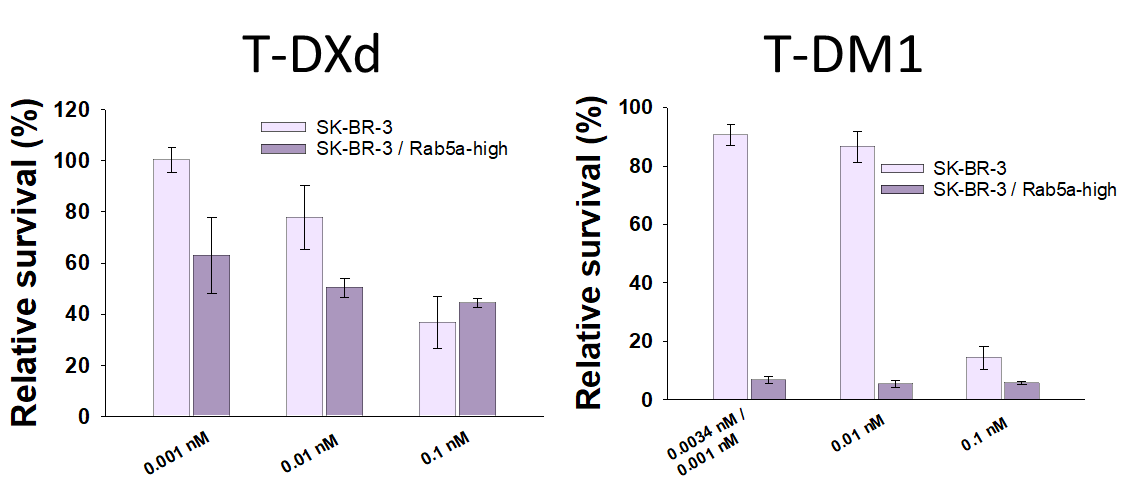


**Figure S4: SK-BR-3 Rab5a-high cell line is more sensitive to T-DXd and T-DM1 treatment compared to SK-BR-3 wild type.** SK-BR-3 was genetically modified to stably express heightened mCherry-Rab5a. Relative viability to three different concentrations is presents with the bars representing the mean of three experiments and the error bar the SD of the mean**.** Raw data of T-DM1 response in Sk-BR-3 wild type is collected from Engebraaten *et.al*.


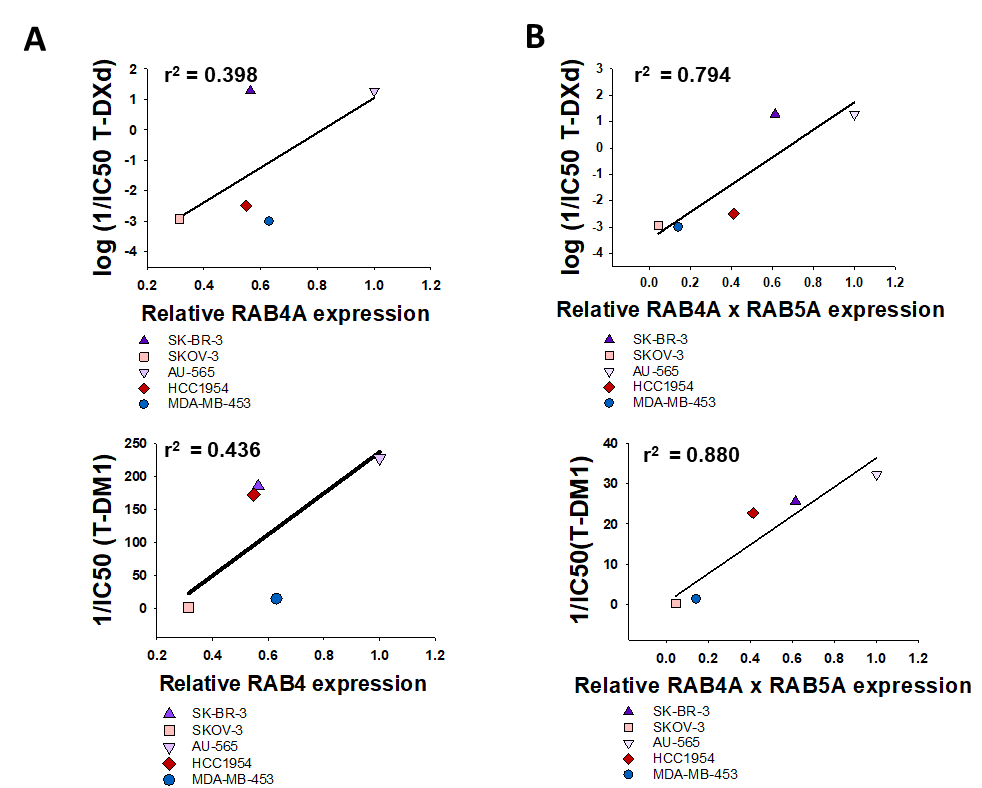


**Figure S5: T-DXd sensitivity correlates the best to RAB4 x RAB5 protein expression**. Linear regression curves between T-DXd and T-DM1 sensitivity and RAB4 expression (A) and RAB4A x RAB5A expression (B). Raw data of RAB5A expression is collected from Engebraaten *et.al*.
